# 512 Gbps/λ dual-polarization thin-film lithium niobate modulators based on an electro-optic equalizer

**DOI:** 10.1515/nanoph-2025-0472

**Published:** 2025-11-25

**Authors:** Jianmin Zhang, Jian Shen, Shuxiao Wang, Zhuoyun Li, Wencheng Yue, Xin Ou, Yan Cai

**Affiliations:** State Key Laboratory of Materials for Integrated Circuits, Shanghai Institute of Microsystem and Information Technology, Chinese Academy of Sciences, Shanghai, 200050, China; University of the Chinese Academy of Sciences, Beijing 100000, China

**Keywords:** thin film lithium niobate, electro-optic modulator, polarization splitter-rotator, Mach-Zehnder interferometer, traveling-wave electrode

## Abstract

Driven by the development of AI applications, optical communication systems experience an exponential surge in the demand for high data rates. Thin-film lithium niobate (TFLN) electro-optic (EO) modulators have been extensively studied and show potential for application in next-generation optical communication systems. In this paper, we present a dual-polarization (DP) TFLN EO modulator integrated based on an EO equalizer, fabricated on the lithium-niobate-on-insulator (LNOI) platform. The device consists of an 11-mm-long forward modulation section and a 4.5-mm-long reverse modulation section. It achieves a half-wave voltage (V_π_) of 4 V for both Y-polarization (Y-pol) and X-polarization (X-pol), and exhibits an on-chip insertion loss of 2.5 dB for Y-pol and 2.8 dB for X-pol at a wavelength of 1,550 nm. A 3-dB EO bandwidth exceeding 110 GHz with low EO roll-off is achieved for both TE and TM modes. Furthermore, the modulator supports a data transmission rate of 512 Gbit/s in 4-level pulse amplitude modulation (PAM4) format, corresponding to 256 Gbit/s per polarization. This work demonstrates a beyond 400 G/λ solution for implementing a high-speed, and large-bandwidth modulator on a conventional LNOI platform.

## 1 Introduction

Optical communication offers a broader channel bandwidth and lower transmission loss compared to electrical communication, making it the preferred choice for data centers and cloud computing infrastructures. High-performance EO modulators, serving as the key components of optical communication systems, enable efficient conversion between electrical and optical signals [1], [2]. EO modulators based on bulk LN, silicon photonics and InP platforms have been extensively investigated and adopted in commercial applications [1], [3], [4], [5], [6], [7], [8]. However, the bulk LN modulator suffers from limitations such as high-wave voltages, small EO bandwidths, and significant losses. These limitations highlight the urgent need for next-generation high-performance electro-optic modulators to sustain the ever-increasing transmission rates required by modern data centers.

TFLN has emerged as an ideal platform for integrating high-speed EO modulators in next-generation optical communication systems, owing to its strong optical field confinement, wide transparent window and large Pockels coefficient (*r*_33_: ∼30 pm/V). These characteristics enable high modulation efficiency, a compact footprint, low insertion loss, and a broad EO bandwidth [9], [10], [11], [12]. LNOI platforms employing quartz or undercut-etched silicon substrate have demonstrated improved EO bandwidth by optimizing the dielectric loss tangent and reducing microwave propagation losses [13], [14], [15], [16], [17], [18]. A TFLN EO modulator with undercut-etched silicon substrate shows a large 3-dB bandwidth of > 70 GHz and a PAM-4 data transmission of 112 Gbit/s [13]. It has been reported that an EO modulator, fabricated on a LN-on-quartz platform, exhibits a large 3-dB bandwidth of > 110 GHz and supports a 95 Gbaud 16QAM data stream [16]. BCB underfill has been employed to further enhance the 3-dB EO bandwidth [18]. However, these approaches for enhancing EO bandwidth and data rates require either complex fabrication processes or non-silicon substrates, which pose challenges for wafer-scale fabrication and large-scale hybrid integration.

Herein, we present an EO modulator with a 3-dB bandwidth of >110 GHz and a data rate of 512 Gbit/s fabricated on an LNOI platform. The EO modulator is co-integrated with an EO equalizer on the same chip, further EO bandwidth [8], [19], [20]. By employing polarization-division multiplexing (PDM), the channel capacity is doubled without additional multi-wavelength light sources [21], [22]. The fabricated 15.5-mm-long TFLN modulator exhibits an ultra-high EO bandwidth in both TE port and TM port, with a 2.5 dB roll-off at 110 GHz. The device exhibits a half-wave voltage V_π_ of 4 V for both Y-pol and X-pol on-chip insertion loss of 2.5 dB for Y-pol and 2.8 dB for X-pol at a wavelength of 1,550 nm, respectively. When transmitting 2 × 256 Gbit/s in PAM4 format, the extinction ratios (ERs) are 2.38 dB for Y-pol and 3.08 dB for X-pol, respectively. Compared to other TFLN MZMs, our modulator achieves a high data rate without employing additional processes or specialized substrates, making this work a promising candidate for next-generation high-capacity, and cost-effective optical communication systems.

## 2 Design of the electro-optic modulator

**Figure 1:**
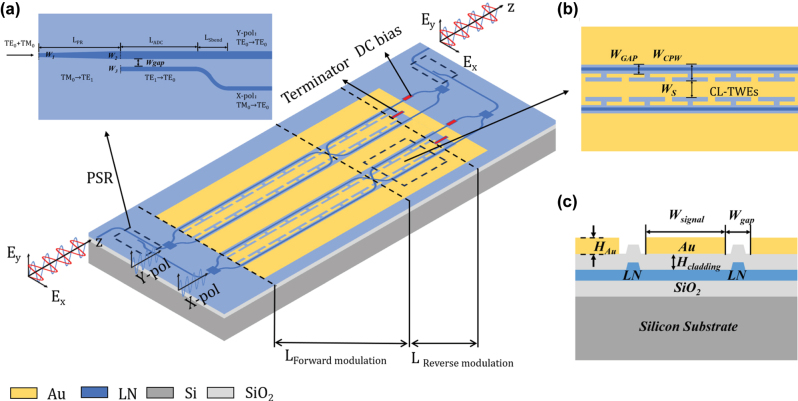
Schematic of the dual-polarization (DP) TFLN MZM based on an EO equalizer. (a) Schematic of the designed polarization splitter rotator. (b) Top view of the capacitive-loaded traveling-wave electrodes and key parameters. (c) Cross-section of the DP-TFLN MZM and key parameters.

Figure 1 shows the schematic and cross-section of DP-TFLN MZM based on an EO equalizer, where the patterned TFLN layer forms the waveguides and coupling, including a pair of polarization splitter rotators (PSRs) for polarization multiplexing, two pairs of 1 × 2 multi-mode interference (MMI) couplers for optical splitting and combining, two crossing waveguides for reversal of modulation direction, and four modulation arms. A pair of mutually parallel Mach–Zehnder interferences, interconnected through PSRs, is formed by the modulation arms and MMI couplers. The GSGSG electrodes in a coplanar waveguide configuration are employed to drive the TFLN waveguides, forming a natural push-pull structure. The DP MZM is fabricated on a LNOI platform. The stack of the LNOI wafer is composed of a 525-um-thick high-resistivity silicon layer (resistivity > 1,000 Ω cm) for reducing substrate losses, a 4.7-μm-thick buried oxide layer, and a 600-nm-thick X-cut TFLN layer. The etching depth of the LN ridge waveguide is chosen to be 300 nm, while the width and the sidewall angle of the waveguide are 1.5 μm and 65°, respectively. A 1-μm-thick silicon dioxide layer is deposited between the TFLN layer and a 1-um-thick Au layer to avoid optical absorption loss from the metal electrodes. An 180-nm-thick nickel chromium (NiCr) layer is employed for the DC biasing and the on-chip terminator.

### 2.1 Polarization splitter rotator

**Figure 2:**
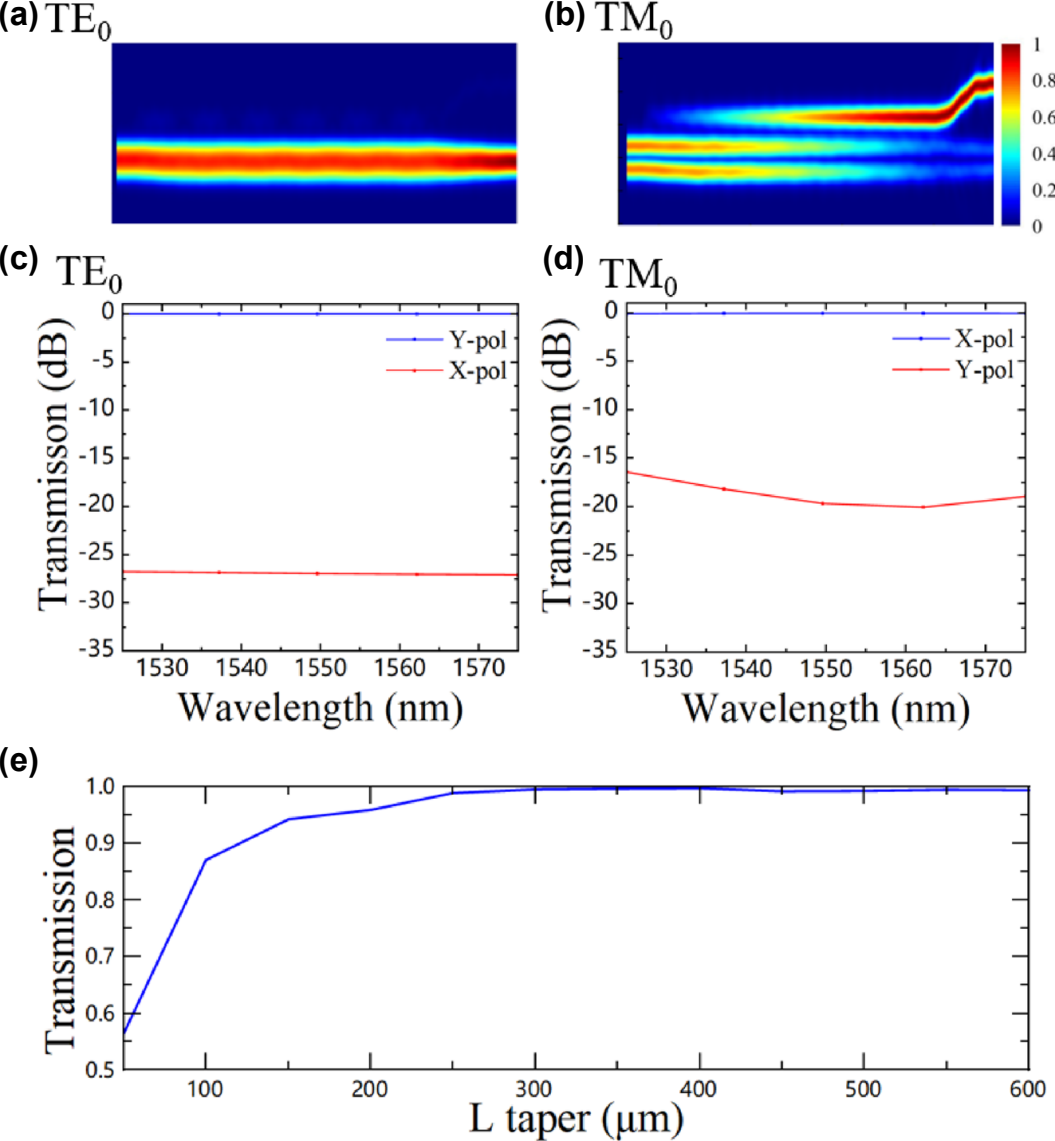
Simulation results of the designed PSR. The mode profile transition in the PSR for (a) TE_0_ (b) TM_0_. The transmission of the PSR from 1,525 nm to 1,575 nm when the input is (c) TE_0_ (d) TM_0_. (e) The mode conversion efficiency versus *L*_taper_ at the wavelength of 1,550 nm.

The maximum EO coefficient of LN is maximized along the *r*_33_ direction, with the X-cut TFLN corresponding to the TE mode polarization state [12], [23]. To achieve maximum couple efficiency for both TE and TM polarization states, a pair of edge couplers is employed for optical input and output coupling. At the input port, the TE mode light is routed to the Y-polarized (Y-pol) port of PSR, and the TM mode is converted to TE mode and routed to the X-polarized (X-pol) port of PSR. After modulation, the beams are recombined through the polarization rotator and combiner (PRC) with both TE and TM polarization states. Figure 1(a) illustrates the proposed on-chip LN PSR. As depicted in Figure 2(a) and (b), the TE component of the input light is routed to the Y-pol, while the TM component is rotated by 90° and output at the X-pol. The LN PSR consists of a cascaded adiabatic taper structure and an adiabatic directional coupler (ADC) structure. The device is aligned along the *Z*-axis of LN. When light propagates along the *Z*-axis of LN, both TE and TM modes exhibit the same refractive index of 2.21. Propagation along the *Z*-direction enables higher efficiency for converting the TM_0_ mode to the TE_1_ mode through mode hybridization compared to the *Y*-direction [24], [25]. As shown in Figure 2(e) according to simulation results, when the adiabatic taper length *L*_taper_ exceeds 400 μm, the mode conversion efficiency exceeds 99.8 % while simultaneously reducing the device footprint and insertion loss. Based on mode matching conditions, when the widths of the two waveguides in the ADC structure are *W*_2_ = 3 μm and *W*_3_ = 1.32 μm, respectively, the TE_1_ mode would be converted to TE_0_ mode and output at the Y-pol port. The distance between the bottoms of the two coupled waveguides, *W*_gap_ are set to 400 nm and 101.7 μm to ensure etching depth. In simulations, this distance is also adopted as the model parameter. The coupling length *L*_ADC_ is set to 101.7 μm for coupling efficiency. As shown in Figure 2(c) and (d), the simulated insertion losses are 0.02 dB at the X-pol and 0.05 dB at the Y-pol, respectively. Meanwhile, the simulated polarization extinction ratio exceeds 20 dB in the C-band.

### 2.2 Capacitive-loaded traveling wave electrode

The performance of traveling wave electrodes (TWEs) directly determines the electro-optic bandwidth of modulators. The relationship between TWEs parameters and the EO response of the modulator is given by [26]:

$$\text{m}\left(\omega \right)=\left|\frac{2Z_{\text{in}}}{Z_{\text{in}}+Z_{G}}\right|\left|\frac{\left(Z_{L}+Z_{0}\right)F_{+}+\left(Z_{L}-Z_{0}\right)F_{-}}{\left(Z_{L}+Z_{0}\right)e^{\gamma_{m}L}+\left(Z_{L}-Z_{0}\right)e^{-\gamma_{m}L}}\right|$$

where *γ*_m_ = α+jβ is the RF complex propagation constant. α is the attenuation coefficient, and β is the phase constant. *Z*_*G*_, *Z*_*L*_ and *Z*_in_ represent the generator impedance, load impedance, and modulator characteristic impedance, respectively. The modulation performance of the TFLN modulator is primarily influenced by three factors: index mismatch between the microwave and optical signals, impedance mismatch, and RF losses of the electrodes [23], [26]. The RF losses of the modulator are mainly caused by conductor losses in the TWEs and dielectric losses in the insulating materials, which are constrained by the material properties of the metal and dielectric layers [18], [20]. The impedance of modulations is largely determined by the ratio of the signal electrode width to the electrode gap. The microwave phase velocity is primarily governed by the permittivity of the dielectric layers surrounding the TWE.

**Figure 3:**
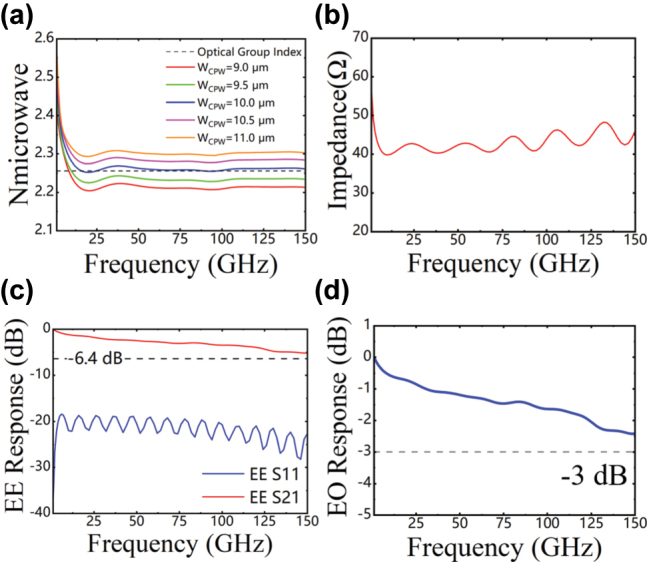
Simulation results of the designed CL-TWEs. (a) Relationship between the width of T-rails electrode *W*_CPW_ and microwave effective index. (b) Characteristic impedance. (c) Simulated S-parameters. (d) EO response with a 43-Ω load resistance.

For capacitively-loaded TWEs (CL-TWEs), the microwave velocity can be flexibly tuned by varying the parameters of capacitively-loaded T-rails. As depicted in Figure 3(a), for a given T-rail electrode gap *W*_GAP_, the gap between the main coplanar waveguide electrodes *W*_CPW_ can be adjusted to minimize the velocity mismatch between the microwave and optical group velocities. A larger *W*_CPW_ leads to an increase in the microwave refractive index. When *W*_CPW_ = 10 μm, the extracted microwave effective refractive index is close to the optical group index (*n*_*g*_ = 2.26 @1,550 nm). As shown in Figure 3(b), the extracted characteristic impedance of our modulator is approximately 43 Ω, slightly lower than the standard 50 Ω. Based on the simulation results, a 43-Ω on-chip resistance is employed in the terminal of the transmission line. Figure 3(c) displays the simulated S-parameters of a TFLN modulator with a 6.5 mm long CL-TWEs (*W*_*s*_ = 40 μm, *W*_gap_ = 4 μm, *W*_cpw_ = 10 μm), showing an electrical 6.4-dB bandwidth of over 150 GHz and good impedance matching with S11 below −17.5 dB. Figure 3(d) presents the simulated 3-dB EO bandwidth based on the extracted RF losses, microwave refractive index, and characteristic impedance, showing that the simulated EO response has a roll-off of less than 3 dB at 150 GHz.

### 2.3 Electro-optic equalizer

**Figure 4:**
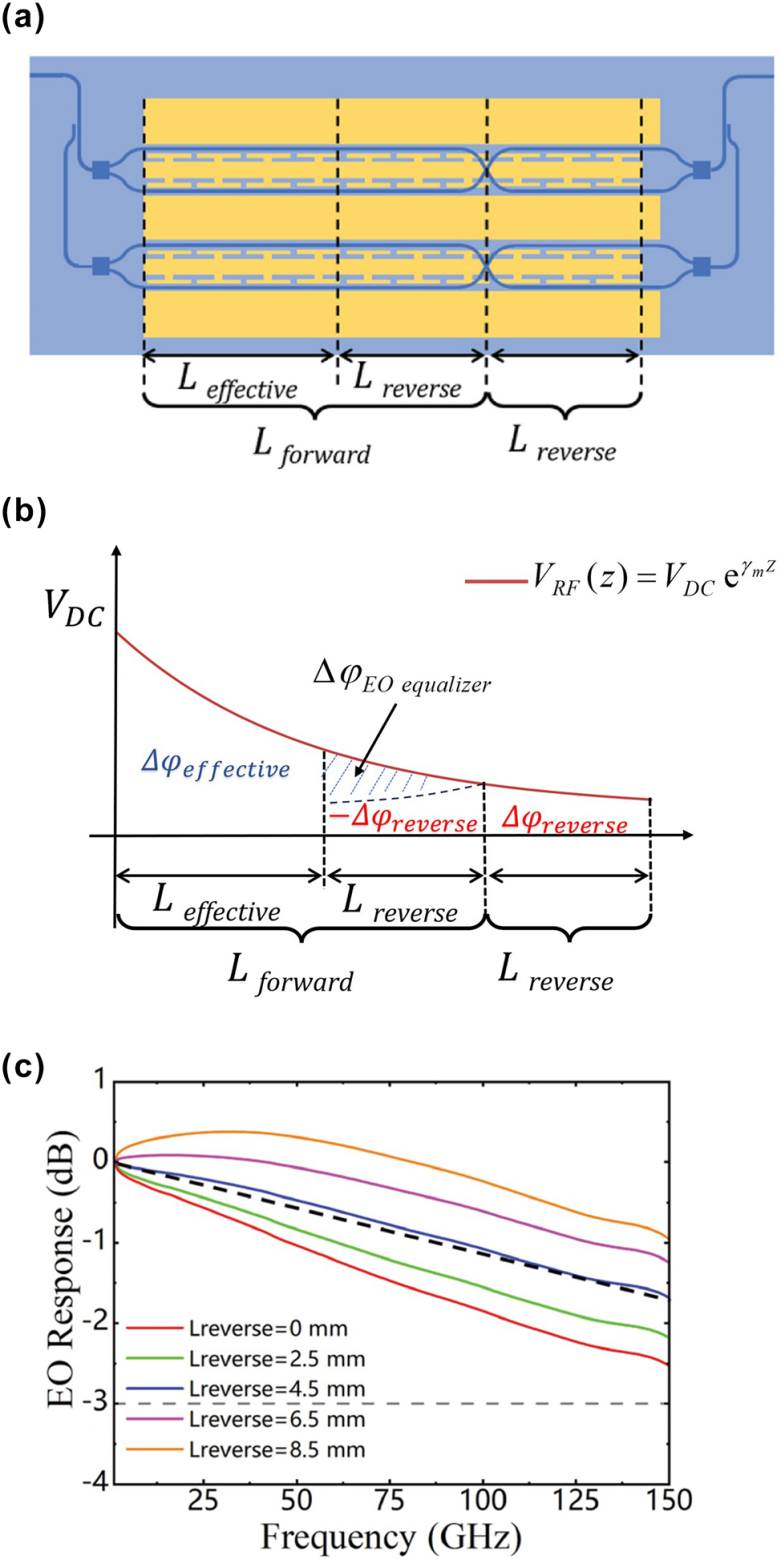
Principle of the proposed MZMs using the EO equalizer. (a) Schematic diagram of the EO equalizer. (b) Phase accumulation and gain versus electrode length of the EO equalizer. (c) Simulated results of the EO response for the EO equalizer modulators, in which *L*_effective_ is 6.5 mm and *L*_reverse_ is from 0 mm to 8.5 mm.

The EO equalizer can enhance the EO bandwidth of EO modulators without additional fabrication processes. Figure 4(a) illustrates a schematic diagram of the designed EO equalizer. The EO modulator based on the EO equalizer can be divided into two sections: the forward modulation section and the reverse modulation section, with lengths denoted as *L*_forward_ and *L*_reverse_. In our design, the polarity reversal of the EO equalizer is achieved using a waveguide crossing. Since the crossing waveguide is much shorter than the transmission line and is a symmetric structure, the modulation of the crossing waveguide region can be neglected [27].

Figure 4(b) presents a physical representation of the EO equalizer to clarify its working principle. In the forward modulation section of the EO equalizer, the cumulative phase change of the optical signal is positive. In the reverse modulation section, the cumulative phase change is negative. In DC conditions, the phase accumulation of the forward modulation section and the reverse modulation section cancel each other. When the drive voltage is the same, an EO modulator with an EO equalizer of length *L*_forward_ + *L*_reverse_ is equivalent to a *L*_effective_ modulator without an EO equalizer. The EO response can be calculated according to the phase accumulation. Clearly, the phase accumulation of a modulator without an EO equalizer is given by the integral of from 0 to *L*_effective_, expressed as: *Δφ*_effective_. The phase accumulation of a modulator with an EO equalizer is given by the integral of the effective voltage from 0 to *L*_forward_ minus the integral of the effective voltage from *L*_forward_ to *L*_forward_ + *L*_reverse_, expressed as: *Δφ*_forward_ – *Δφ*_reverse_. Since the *V*_RF_ attenuates exponentially with the propagation length, the accumulated phase of the EO equalizer modulator is larger than *Δφ*_effective_. *Δφ*_EOqualizer_ is defined as the additional phase accumulation achieved by a modulator with an EO equalizer compared to the one without an EO equalizer. The EO response of modulators with an EO equalizer can be given as follows (assuming a matched load and generator (*Z*_*L*_ = *Z*_*G*_ = *Z*_in_) and a matched optical and microwave index) [20]:

$$\begin{array}{cl} & m{\left(\omega \right)}_{\text{EOqualizer}}\\ & \;=\left|\frac{\Delta \varphi_{\text{forward}}\left(\omega \right)-\Delta \varphi_{\text{reverse}}\left(\omega \right)}{\Delta \varphi_{\text{effective}}\left(0\right)}\right|\;\\ & \;=\left|\frac{\int_{0}^{L_{\text{forward}}}V_{RF}\left(\omega ,z\right)dz-\int_{L_{\text{forward}}}^{L_{\text{forward}}+L_{\text{reverse}}}V_{RF}\left(\omega ,z\right)dz}{\int_{0}^{L_{\text{effective}}}V_{DC}dz}\right| \end{array}$$

Herein, *V*_*DC*_ is the input voltage, *V*_*RF*_ is *V*_*DC·*_ *e* ^*γmz*^. *γ*_*m*_ *=α+jβ* is the RF complex propagation constant. The parameters for the EO equalizer are set as follows: Firstly, the *L*_effective_ of 6.5 mm is chosen to achieve a half-wave voltage below 5 V, ensuring that the data transmission performance of EOMs is not limited by a high half-wave voltage. As shown in Figure 4(c), we simulate the EO response of the EO equalizer MZMs with *L*_effective_ of 6.5 mm and *L*_reverse_ of 0–8.5 mm. The *L*_effective_ and *L*_reverse_ are designed to be 6.5 mm and 4.5 mm, respectively, to achieve a linear roll-off EO response which ensures higher signal fidelity and more stable eye diagrams [28], [29]. The modulator with an EO equalizer exhibits a roll-off of only 1 dB across the frequency range from 1 GHz to 150 GHz, demonstrating its effectiveness in reducing the EO loss of the device.

## 3 Device fabrication and characterization

**Figure 5:**
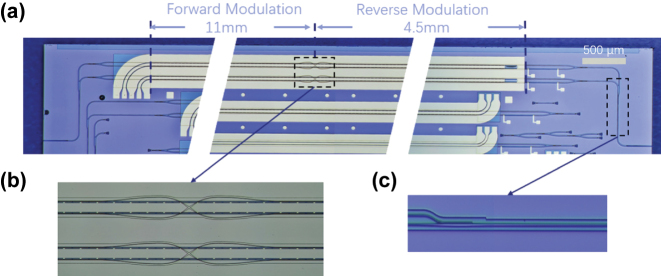
Micrograph of (a) the device with an 11-mm-long forward modulation section and a 4.5-mm-long reverse section. (b) the CL-TWEs and the waveguide crossing. (c) the PSR.

As shown in Figure 5(a), we fabricated a 15.5-mm-long DP-TFLN MZM based on the EO equalizer on a 600-nm-thick X-cut LNOI platform using deep ultraviolet (DUV) lithography. Figure 5(b) and (c) shows the optical microscope image of the CL-TWEs, waveguide crossing and PSRs. The device consists of an 11-mm-long forward modulation section and a 4.5-mm-long reverse modulation section. The parameters of CL-TWEs are set as *W*_*s*_ = 40 μm, *W*_gap_ = 4 μm, *W*_cpw_ = 10 μm. The TFLN ridge waveguides, with a width of 1.5 μm and a thickness of 300 nm, were fabricated via reactive-ion etching (RIE) with argon. The 1-μm-thick gold electrodes are fabricated using lift-off techniques. At the terminals of the CL-TWEs, NiCr alloy is employed as on-chip termination resistors and DC bias for avoiding DC-drift and reducing energy consumption. Since the on-chip terminations were utilized in the DP-TFLN MZM, a 6.5-mm-long modulator without the EO equalizer and termination is fabricated to characterize the modulation efficiency, microwave index, and RF losses. This reference device features the same electrode and waveguide parameters as the MZM with an EO equalizer.

**Figure 6:**
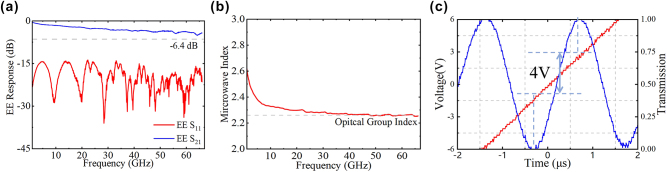
Measured result of the 6.5-mm-long MZM (*W*_*s*_ = 40 μm, *W*_gap_ = 4 μm, *W*_cpw_ = 10 μm). (a) Electro-electrical (EE) S_11_ and S_21_. (b) Extracted microwave index. (c) Half-wave voltage at 1,550 nm.

Figure 6(a) plots the S-parameters of a 6.5-mm-long CL-TWE MZM, measured using a 67 GHz performance network analyzer (PNA, Keysight N5227B) and a pair of 67 GHz GSG microwave probes. Within the range from 10 MHz to 67 GHz, the EE S_21_ remains above −4.5 dB, while the return loss is better than −14 dB, indicating good impedance matching of the transmission line. As shown in Figure 6(b), the device exhibits a half-wave voltage of 4 V. We extracted the microwave index according to the phase of the EE S_21_, as shown in Figure 6(c). The microwave index is close to 2.25 at 67 GHz, demonstrating the good index matching between microwave and optical wave.

The insertion loss of the 15.5-mm-long EO equalizer MZM is characterized using a 2.5-µm MFD fiber at 1,550 nm, with an on-chip insertion loss of 2.5 dB for Y-pol and 2.8 dB for X-pol. The edge-coupling loss is 1.29 dB per facet, while the 1 × 2 MMI and waveguide crossing contribute 0.15 dB and 0.2 dB, respectively. The insertion loss of edge couplers is obtained by subtracting the transmission loss of waveguides from two edge couplers connected by a straight waveguide. The insertion loss of crossing waveguides and MMIs is derived by subtracting the insertion loss of the reference grating from the cascaded structure. The insertion loss mainly originates from edge coupling, which is caused by the mode field mismatch between the submicron-sized waveguide and the optical fiber. This mismatch can be effectively reduced by employing a trident coupler [30].

**Figure 7:**
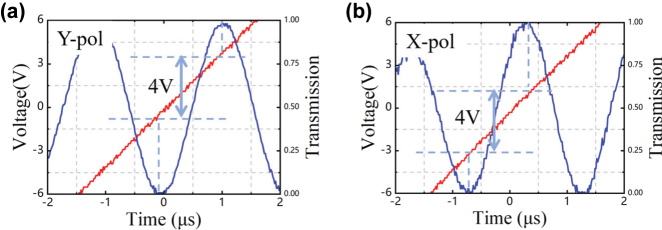
Half-wave voltage measured result of the 15.5 -mm-long MZM at 1,550 nm. (a) Y-pol. (b) X-pol.

As shown in Figure 7(a) and (b), the half-wave voltages of the device are 4.0 V for both Y-pol and X-pol. The half-wave voltage of the EO equalizer is the same as that of a device without an EO equalizer under the same effective modulation length, indicating the half-wave voltage is not influenced by the EO equalizer [20].

**Figure 8:**
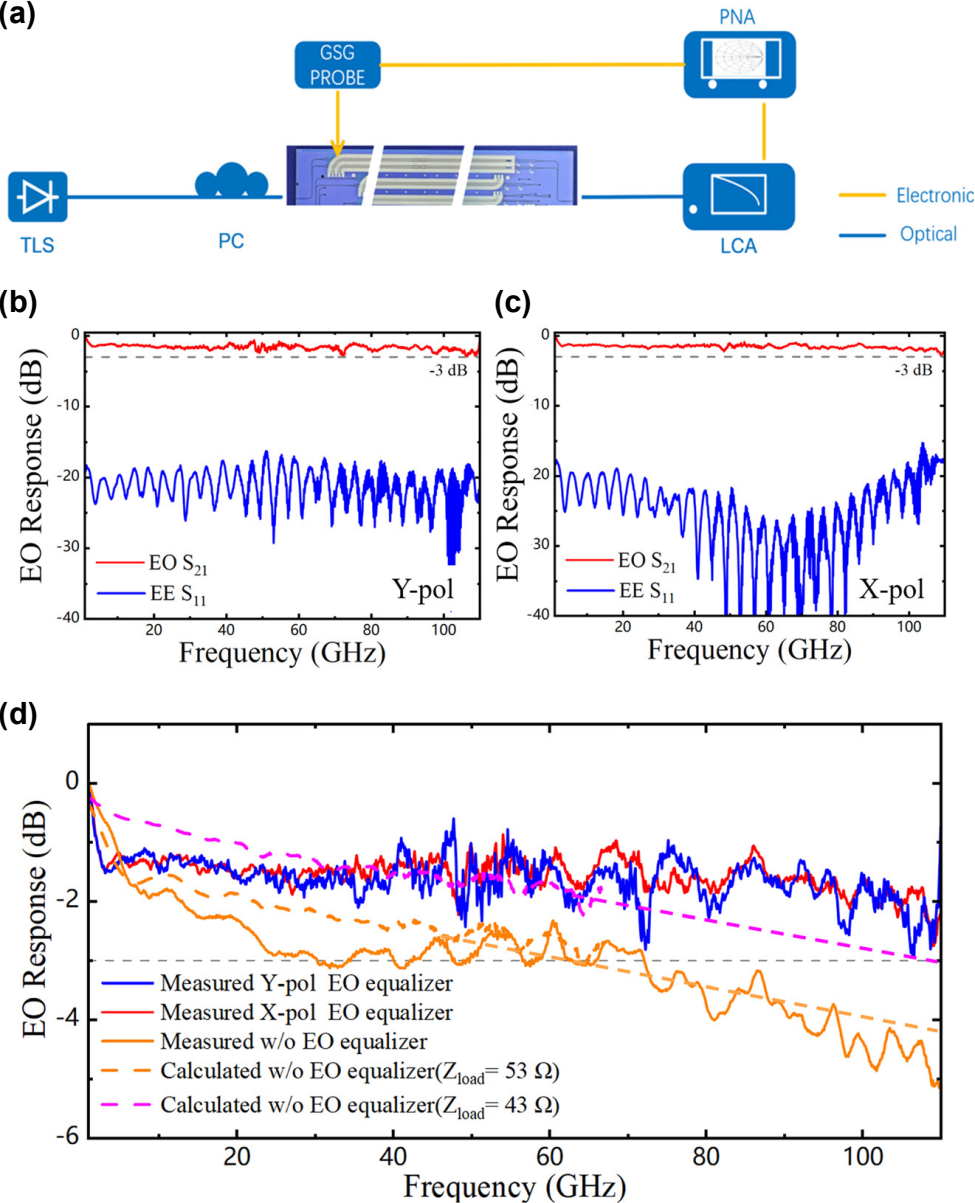
Measured results of the EO response for the MZMs at 1550 nm. (a) Schematic of the EO response measurement setup. Measured EO S21 and S11 for (b) Y-pol and (c) X-pol. (d) Measured EO S_21_ for Y-pol (red line), X-pol (blue line), and the CL-TWEs MZM without the equalizer (orange line). Calculated EO S_21_ for MZM without the equalizer with on-chip resistances of 53 Ω (dashed orange line) and 43 Ω (dashed pink line).

Figure 8(a) illustrates the setup for the EO response measurement. The EO responses of a 15.5-mm-long DP-TFLN MZM based on an EO equalizer and a 6.5-mm-long conventional TFLN MZM are measured using a 110 GHz vector network analyzer (VNA), a 110 GHz LCA and a 110 GHz GSG microwave probe. As exhibited in Figure 8(b) and (c), the EO loss is less than 3 dB, and the return loss is below −18 dB within the 110 GHz range, demonstrating the low EO loss and good impedance matching in the transmission line. Figure 8(d) presents a comparative analysis of the EO responses obtained with and without the EO equalizer for both Y-pol and X-pol, while maintaining the same effective modulation length. All curves of EO S_21_ exhibit a steep roll-off at low frequencies, which is attributed to the measured on-chip resistance of 53 Ω, higher than the designed 43 Ω [23], [31]. Based on the EE response of the 6.5-mm device and the measured microwave refractive index, we calculated and predicted the EO bandwidths for on-chip resistances of 43 Ω and 53 Ω, which agree well with the measurements. The Y-pol and X-pol exhibit similar EO bandwidth curves and EO loss. Compared to the EO response of the reference device without the equalizer, it is evident that the EO equalizer effectively enhances the EO response, reducing the EO loss by approximately 2.5 dB at 110 GHz. Based on the measured voltage and EO bandwidth, the EO equalizer effectively mitigates the inherent trade-off between half-wave voltage and EO bandwidth.

**Figure 9:**
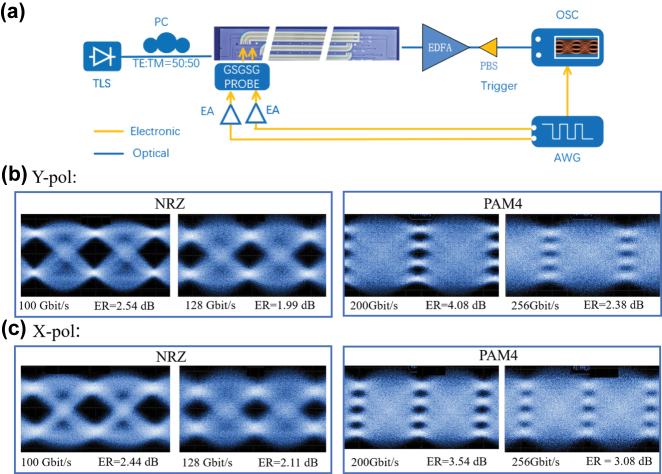
High-speed data transmission measurements of the DP TFLN MZM with non-return-to-zero (NRZ) and PAM4 at the wavelength of 1,550 nm. (a) The measurement setup. The eye diagram results of (b) Y-pol and (c) X-pol.

The high-speed data transmission of the DP TFLN MZM based on an EO equalizer is verified by eye-diagram measurements, with the experimental setup illustrated in Figure 9(a). At the transmitter, a 256 GSa/s arbitrary waveform generator (AWG, Keysight M8199A) generates a (2^15^-1)-bit pseudo-random binary sequence (PRBS), which is subsequently mapped onto NRZ and PAM4 symbols. The transmission rates are mainly constrained by the bandwidths of the electrical amplifier (55 GHz), OSC (65 GHz), and RF probe (67 GHz).

In Table 1, the performance of the proposed EO-equalized modulator is compared with state-of-the-art MZMs on different substrates. The demonstrated modulator exhibits excellent performance in terms of the EO bandwidth and data transmission rates. Fabricated on a silicon-based LNOI platform, the device offers simpler fabrication flow and lower manufacturing cost.

**Table 1:** Performance of TFLN modulators in the C-band.

References	V_π_(V)	Bandwidth (GHz)	Data rate (Gbit/s)	Substrate
[9]	1.4	45	210 (PAM8)	Silicon
[32]	5.1	>70	112 (PAM4)	Silicon
[33]	3.4	67	N/A	Quartz
[13]	1.7	70	168 (PAM8)	Undercut-etched Silicon
[34]	2.18	>67	112 (PAM4)	Backside-holes Silicon
[17]	3.2	>67	N/A	Quartz
[18]	1.9	>110	390 (PAM8)	Quartz + BCB
**This work**	4.0	**>110**	**512 (PAM4)**	**Silicon**

The RF signal is then amplified by a 55-GHz electrical amplifier (SHF S807C, 55 GHz) and delivered to the device via a 67-GHz ground-signal-ground-signal-ground (GSGSG) RF probe. A trigger signal from the AWG is fed into an electrically sampled oscilloscope (OSC, Keysight N1000A, 65 GHz) as the clock reference. A polarization controller is used to adjust the polarization components of the input light. The polarization components of input light are monitored through a polarization beam splitter (PBS, Thorlabs PFC1550). When the optical powers along the fast and slow axes of the PBS are equal, the TE and TM polarization components with a power ratio of 50:50 are input to the DP-TFLN MZM. After being modulated and amplified by an erbium-doped fiber amplifier (EDFA), the dual-polarization signals are separated using a PBS, and then fed into the photodetector (PD) of the OSC to obtain eye diagrams for the Y-pol and X-pol.

As depicted in Figure 9(b) and (c), the clearly opened eye diagrams of 100 Gbit/s and 128 Gbit/s NRZ signals, as well as 200 Gbit/s and 256 Gbit/s PAM4 signals, are measured under both Y-pol and X-pol. The ERs for PAM4 and NRZ signal are defined as the average optical powers of the highest level divided by the lowest level. The ERs under Y-pol are 2.54 dB and 1.99 dB for the 100 Gbit/s and 128 Gbit/s NRZ signals, and 4.08 dB and 2.38 dB for the 200 Gbit/s and 256 Gbit/s PAM4 signals, respectively. Under X-pol, the corresponding ERs are 2.44 dB and 2.11 dB for the NRZ signals, and 3.54 dB and 3.08 dB for the PAM4 signals, respectively. The measured bit-error rates (BERs) for the NRZ signals at 80 Gbit/s, 100 Gbit/s, and 128 Gbit/s are 3 × 10^−8^, 9 × 10^−7^, and 9 × 10^−3^.

## 4 Conclusions

In this work, a high-performance DP-TFLN MZM with an EO equalizer is designed and fabricated in an LNOI platform. Regarding the overall transmission rate, the PDM scheme proposed in this work provides a total data rate equal to the combined rates of two modulators, while maintaining the same number of input/output ports and electrical drivers. Due to the EO-equalizer effect, the fabricated 15.5-mm-long device based on an EO equalizer shows a 3-dB EO bandwidth exceeding 110 GHz, indicating an improvement of over 60 GHz compared to conventional modulators. Furthermore, the device exhibits a low half-wave voltage of 4.0 V and low on-chip insertion loss of 2.5 dB for Y-pol and 2.8 dB for X-pol. The modulator supports a 128 GBaud PAM4 transmission for both Y-pol and X-pol. A data transmission rate of 512 Gbit/s has been successfully demonstrated. The EO equalizer and PDM techniques can boost the data transmission rate on an LNOI platform without additional processes which are well-suited for applications in next-generation data center and coherent optical communication networks. In future work, we will extend the folded structure to EO equalizer modulators to reduce the footprint of devices.
